# Current challenges and future opportunities in patient‐focused management of hereditary angioedema: A narrative review

**DOI:** 10.1002/clt2.12243

**Published:** 2023-05-20

**Authors:** Anete S. Grumach, Noga Gadir, Aharon Kessel, Ashley Yegin, Inmaculada Martinez‐Saguer, Jonathan A. Bernstein

**Affiliations:** ^1^ Clinical Immunology, Faculdade de Medicina Centro Universitario Faculdade de Medicina ABC (FMABC) Santo Andre Brazil; ^2^ Takeda Development Center Americas, Inc. Lexington Massachusetts USA; ^3^ Division of Allergy and Clinical Immunology, Bnai Zion Medical Centre Technion Faculty of Medicine Haifa Israel; ^4^ HRZM Hemophilia Center Rhein Main Mörfelden‐Walldorf Germany; ^5^ Division of Rheumatology, Allergy and Immunology University of Cincinnati College of Medicine and Bernstein Clinical Research Center Cincinnati Ohio USA

**Keywords:** C1 inhibitor, guidelines, hereditary angioedema, management, quality of life

## Abstract

Patients with hereditary angioedema (HAE) experience a high burden of disease due to unpredictable, painful, disfiguring, and potentially life‐threatening HAE attacks. Multiple HAE‐specific medications for the on‐demand treatment, short‐term and long‐term prophylaxis of HAE attacks have entered the market in recent years; however, the availability and access to these medications may vary between different countries. For this review, PubMed and EMBASE databases were searched for guidelines, consensus statements, and other publications on HAE management as well as publications on quality of life in patients with HAE. The current guidelines and recent literature on HAE management in specific countries are summarized with the aim to highlight the similarities and differences between guideline recommendations and the country‐specific clinical practice. Improvement in quality of life, which is a key goal in HAE management, is also discussed and the country‐specific trends are highlighted. Finally, the ways to achieve a more patient‐centric approach to HAE management within the framework set by the clinical management guidelines are examined.

## 1 INTRODUCTION

Hereditary angioedema (HAE) is a rare, potentially life‐threatening genetic disorder.^1^ The estimated prevalence of HAE for the overall population is 1:50,000.^1^ Most of the cases of HAE are due to mutations in the *SERPING1* gene coding for C1 inhibitor (C1‐INH), leading to a deficiency (HAE Type 1) or dysfunction (HAE Type 2) of C1‐INH.^2^, ^3^ Less frequently, patients with HAE may have normal C1‐INH quantity and function and no mutation in the *SERPING1* gene – this is known as HAE with normal C1 inhibitor (nC1‐INH‐HAE) and is caused by mutations in one of several different genes, including but not limited to genes coding for coagulation factor XII, plasminogen, and angiopoietin‐1 (Table 1).^3^

**TABLE 1:** Known genes associated with HAE.^4^, ^9^, ^21^

HAE type	Affected gene	Gene product
HAE Type 1	*SERPING1*	C1‐INH (mutations leading to deficiency of protein)
HAE Type 2	*SERPING1*	C1‐INH (mutations leading to dysfunction of protein)
nC1‐INH‐HAE	*F12*	Coagulation factor XII
	*PLG*	Plasminogen
	*KNG1*	Kininogen 1
	*ANGPT1*	Angiopoietin 1
	*HS3ST6*	Heparan sulfate‐glucosamine 3‐O‐sulfotransferase 6
	*MYOF*	Myoferlin

Abbreviations: C1‐INH, C1 inhibitor; HAE, hereditary angioedema; nC1‐INH‐HAE, hereditary angioedema with normal C1 inhibitor.

Clinically, HAE presents as recurrent, unpredictable attacks of swelling without urticaria affecting skin and subcutaneous tissues, abdominal organs, and/or the upper airway, and the clinical presentation of nC1‐INH‐HAE is thought to be similar to that of HAE Type 1/2.^2^, ^4^, ^5^ The clinical presentation of HAE Type 1/2 and nC1‐INH HAE is also similar to that of acquired recurrent angioedema without urticaria, different types of which include acquired angioedema with C1‐INH deficiency; angiotensin‐converting enzyme inhibitor (ACEi) associated acquired angioedema; and acquired idiopathic histaminergic or non‐histaminergic angioedema.^6^

HAE attacks place a significant burden on the patient. Subcutaneous attacks can cause substantial short‐term disability and disfigurement; abdominal attacks are painful and are sometimes mistaken for acute abdominal emergencies such as appendicitis, leading to unnecessary surgical procedures; and laryngeal attacks can cause death by asphyxiation.^2^

Although HAE cannot be cured, pharmacologic management of HAE is available, with the goals of achieving disease control and ensuring that patients are not experiencing limitations from HAE symptoms.^4^, ^7^, ^8^, ^9^ The pharmacologic treatment of HAE can be divided into three categories: on‐demand treatment, short‐term prophylaxis (STP), and long‐term prophylaxis (LTP).^4^ On‐demand treatment is the treatment of attacks as soon as they become apparent and is used to reduce the severity, duration, and associated morbidity of HAE attacks and prevent potential mortality.^8^, ^9^, ^10^ STP is used to minimize the risk of HAE attack occurring in situations where higher risk of HAE attacks is expected, such as before medical or dental procedures. The medication for STP of HAE attacks may be administered either just before or up to several days before the predictable trigger, depending on the type of medication used for STP.^4^, ^9^, ^10^ Finally, LTP of HAE attacks is the routine use of medication to reduce the burden of disease by preventing occurrence of HAE attacks.^4^, ^10^

The armamentarium for LTP of HAE attacks has considerably expanded since 2017, with a subcutaneous formulation of plasma‐derived C1‐INH (pdC1‐INH) approved in the United States in 2017 and the European Union (EU; approval date varies per country),^11^, ^12^ subcutaneous monoclonal antibody against plasma kallikrein lanadelumab approved both in the United States and the EU in 2018,^13^, ^14^ and oral kallikrein inhibitor berotralstat approved in the United States in 2020 and in the EU in 2021.^15^, ^16^

New and upcoming treatment options allow treatments to be tailored to individual patients. The aim of this narrative review is to discuss the challenges to patient‐centric approaches for HAE management and the opportunities in this field within the context of current treatment guidelines.

## 2 METHODS

To assess the current knowledge of HAE management, PubMed and EMBASE databases were searched for consensus statements and guidelines on HAE, as well as for studies from 2016 to 2021 reporting on the management of patients with HAE and/or quality of life (QoL) in patients with HAE.

### 2.1 Guideline recommendations on clinical management of HAE

**FIGURE 1:**
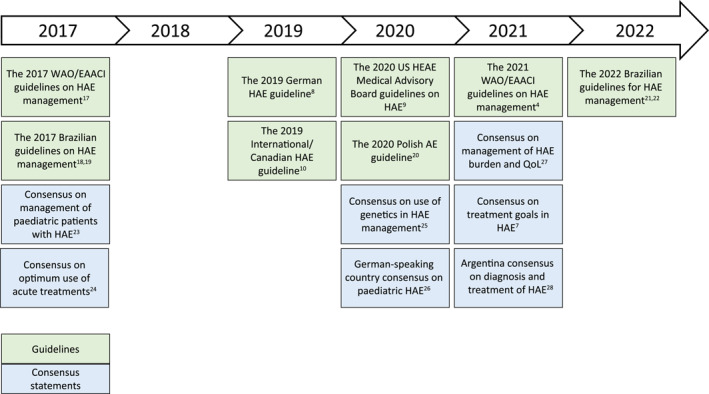
Summary of HAE guidelines and consensus papers published in 2017–2022. AE, angioedema; EAACI, European Academy of Allergy and Clinical Immunology; HAE, hereditary angioedema; HAEA, Hereditary Angioedema Association; QoL, quality of life; WAO, World Allergy Organization.

In recent years, multiple international^4^, ^10^, ^17^ and national^8^, ^9^, ^18^, ^19^, ^20^, ^21^, ^22^ guidelines on the management of HAE as well as several consensus papers^7^, ^23^, ^24^, ^25^, ^26^, ^27^, ^28^ on different aspects of HAE management were published (Figure 1). Overall, these guidelines and consensus papers provide similar recommendations for the pharmacological treatment of HAE Type 1/2 (see Table 2 for detailed summary).

**TABLE 2:** Summary of recent guidelines and consensus statements on HAE management in all patients with HAE Type 1/2.

Guideline	On‐demand treatment	STP	LTP
International guidelines			
The 2019 International/Canadian HAE guideline^10^	Effective therapies should be used for acute treatment of HAE attacks. IV pdC1‐INH, icatibant, ecallantide, and IV rhC1‐INH are effective therapies for acute treatment of attacks. Frozen plasma could be used for acute treatment of HAE attacks if other recommended therapies are not available. Attenuated androgens and tranexamic acid should not be used for acute treatment of HAE attacks.	STP should be considered prior to known patient‐specific triggers and for any medical, surgical, or dental procedures. All patients should have 2 doses of appropriate on‐demand therapy immediately available during and after any procedure. IV pdC1‐INH should be used for STP. Preprocedural prophylaxis with IV pdC1‐INH (20 U/kg) is recommended within an hour before a procedure. Attenuated androgens or frozen plasma may be considered for STP when pdC1‐INH is not available, and if acute treatments for HAE are not available. Danazol (2.5–10 mg/kg per day, maximum 600 mg per day) can be considered for starting 5 days before and continued 2–3 days after the anticipated procedure or trigger. Tranexamic acid should only be used for STP if other therapies are not available. Lanadelumab and SC C1‐INH should not be used for STP.	SC C1‐INH or lanadelumab should be used as first‐line therapy for LTP of HAE attacks. Attenuated androgens and antifibrinolytics should not be used as a first‐line therapy for LTP of HAE attacks, but they may be considered in patients who have already obtained benefit from their use or who have difficulty obtaining first‐line options.
The 2021 WAO/EAACI guidelines on HAE management^4^	IV C1‐INH, ecallantide, or icatibant are recommended as first‐line therapies for on‐demand treatment of HAE attacks. Where first‐line therapies are not available, attacks should be treated with SDP. If SDP is not available, attacks should be treated with FFP where safe supply is available. Use of antifibrinolytics or androgens for on‐demand treatment of HAE attacks is advised against.	It is recommended to consider STP before medical, surgical, or dental procedures, and exposure to HAE attack‐inducing events. IV pdC1‐INH is recommended as first‐line STP. Preprocedural prophylaxis with IV pdC1‐INH should be used as close as possible to the start of procedure. Dosage of IV pdC1‐INH has yet to be fully established; 1000 IU or 20 U/kg are most used. rhC1‐INH could be considered for STP if IV pdC1‐INH is not available. FFP may be used for STP, but is not as safe as pdC1‐INH. Attenuated androgens have been recommended in the past, but IV pdC1‐INH is considered the STP treatment of choice. Androgens for preprocedural prophylaxis are used for 5 days before and 2–3 days after the procedure. Tranexamic acid for preprocedural prophylaxis is not recommended by most guideline experts.	pdC1‐INH, lanadelumab, and berotralstat are recommended as options for first‐line LTP. Attenuated androgens are recommended only as second‐line LTP. Antifibrinolytics are not recommended for LTP.
National guidelines			
The 2019 German HAE guideline^8^	pdC1‐INH, icatibant, and rhC1‐INH are effective for the acute treatment of HAE attacks in adults. FFP should only be considered if none of the above‐mentioned treatments are available.	Patients should receive C1‐INH as STP before all interventions associated with mechanical effects on the upper respiratory and digestive tracts. C1‐INH should be administered 1 h prior to procedure.	C1‐INH can be used for LTP. Attenuated androgens are not approved in Germany and need to be obtained via international pharmacy. Danazol and oxandrolone are used, stanozolol is no longer available. Antifibrinolytics (epsilon‐aminocaproic acid and tranexamic acid) have proven effective in patients with HAE Type 1/2. Progestins are not approved, but they can be helpful for treatment of women with HAE Type 1/2. Progestins should not be combined with androgens or tranexamic acid.
The 2020 US HAEA Medical Advisory Board guidelines for the management of HAE^9^	pdC1‐INH, rhC1‐INH, ecallantide, and icatibant are FDA‐approved therapies for acute treatment of HAE attacks. FFP can be used for HAE attack treatment if none of the above‐mentioned medications are not available. SDP may be safer than FFP. Anabolic androgens and antifibrinolytics have no role in on‐demand treatment.	A single dose of pdC1‐INH or a course of anabolic androgens can be used for STP. pdC1‐INH (20 IU/kg) can be given 1–12 h before the stressor. Anabolic androgens (danazol 400–600 mg/day) can be started 5–7 days before and continued for 2–5 days after the procedure. FFP can be used in the event when pdC1‐INH is not available and there is insufficient time for a course of anabolic androgens.	First‐line therapies for LTP are IV pdC1‐INH, SC pdC1‐INH, and lanadelumab. Second‐line therapies include anabolic androgens and antifibrinolytics (tranexamic acid or epsilon‐aminocaproic acid). Second‐line therapies should be used when first‐line therapies are not available or when patients will only accept oral therapy.
The 2020 Polish AE guidelines^20^	IV pdC1‐INH or rhC1‐INH should be used for the treatment of acute HAE attacks. Icatibant is an alternative for C1‐INH for treatment of acute HAE attacks. FFP may be used for acute treatment of HAE attack if pdC1‐INH and icatibant are not available.	STP should be administered before medical procedures involving mechanical compression or injuries in the oral cavity, throat, or larynx, or procedures that can induce an attack. pdC1‐INH is the therapy of choice for STP. pdC1‐INH for STP should be used at least 6 h before the procedure. FFP can be used if pdC1‐INH is unavailable.	First choice LTP is pdC1‐INH or lanadelumab. Androgen derivatives (danazol, stanazolol) are an alternative for LTP. Antifibrinolytic drugs are given when other options are contraindicated or unavailable.
The 2022 Brazilian guidelines for HAE^22^	Icatibant and pdC1‐INH are first‐line treatments. In the absence of first‐line treatments, FFP may be prescribed.	Used before procedures such as dental treatment or endoscopy. pdC1‐INH (first‐line treatment) may be used. STP with pdC1‐INH (20 U/kg) should be used 1–6 h before the procedure. If there is no access to pdC1‐INH, attenuated androgens (second‐line treatment) are suggested. STP with danazol (2.5–10 mg/kg up to 200 mg per 8–12 h) should be used for 5 days before and 2–3 days after the procedure. In the absence of pdC1‐INH, FFP may be prescribed. STP with FFP (10 ml/kg) should be used 1–6 h before the procedure.	C1‐INH (SC [preferably] or IV) or lanadelumab are first‐line treatments. Attenuated androgen danazol, at maximum dose of 200 mg/day, is a second‐line treatment. As only danazol is available in Brazil's Unified Health System, second‐line treatment with attenuated androgens should be tried first. First‐line treatments (lanadelumab or C1‐INH) are considered according to response to androgen treatment, contraindication, or adverse events due to androgen use.
Consensus statements			
Consensus on acute treatments for HAE^24^	pdC1‐INH, rhC1‐INH, ecallantide, and icatibant are effective acute treatments.		
Argentine consensus on the diagnosis and treatment of HAE^28^	No summary is provided as the guidelines are only published in Spanish.		

*Note*: Only the current versions of guidelines available in English language were summarized.

Abberviations: AE, angioedema; C1‐INH, C1 inhibitor; EAACI, European Academy of Allergy and Clinical Immunology; FDA, Food and Drug Administration; FFP, fresh frozen plasma; HAE, hereditary angioedema; HAEA, Hereditary Angioedema Association; IV, intravenous; LTP, long‐term prophylaxis; pdC1‐INH, plasma‐derived C1 inhibitor; rhC1‐INH, recombinant human C1 inhibitor; SC, subcutaneous; SDP, solvent detergent–treated plasma; STP, short‐term prophylaxis; WAO, World Allergy Organization.

One of the recommendations is to consider on‐demand treatment for all HAE attacks, with special emphasis placed on treating any HAE attacks affecting or potentially affecting the airway.^4^, ^8^, ^9^, ^10^, ^22^ It is recommended that all patients with HAE should have at least two doses of on‐demand medication easily accessible to them at all times.^4^, ^8^, ^9^, ^20^, ^22^ C1‐INH, icatibant, and ecallantide (where available) are the recommended therapies for on‐demand treatment of HAE attacks; fresh frozen plasma (FFP) is an option only when these treatments are not available.^4^, ^8^, ^9^, ^10^, ^20^, ^22^, ^24^ Attenuated androgens and antifibrinolytics are not recommended for on‐demand treatment of HAE attacks.^4^, ^9^, ^10^

The guidelines recommend to consider STP before medical, surgical, or dental procedures, as well as before events that could cause HAE attacks.^4^, ^8^, ^9^, ^10^, ^20^, ^22^ In particular, procedures involving mechanical impact to upper respiratory or digestive tracts, including but not limited to: facial surgery; invasive dental treatment (dental surgery, tooth extraction); bronchoscopy; esophagogastroduodenoscopy; tonsillectomy; and surgical procedures requiring endotracheal intubation, may warrant consideration for STP.^4^, ^9^, ^10^, ^22^ As there is a lack of data regarding the risk of specific procedures, expert clinical judgment and individualized risk assessment are important when making decisions of when to administer STP.^4^, ^9^, ^10^, ^22^ The considerations on when to administer STP may include the degree of physical trauma involved with a specific procedure and whether the individual patient has history of HAE attacks under similar circumstances.^9^, ^10^, ^22^ Additionally, the 2021 WAO/EAACI guidelines highlighted that the need for STP may be different in patients who already are receiving LTP of HAE attacks, although no specific recommendations were given due to lack of data.^4^ C1‐INH is the recommended treatment for STP of HAE attacks.^4^, ^8^, ^9^, ^10^, ^20^, ^22^ FFP and androgens can be used as a second‐line therapy for STP, although the 2020 US HAE guidelines include androgens among first‐line options for STP of HAE attacks, along with C1‐INH.^4^, ^9^, ^20^, ^22^ Furthermore, on‐demand treatment should be available regardless if STP was administered or not.^4^, ^10^ Additional considerations may apply in countries where access to C1‐INH is challenging, thus limiting C1‐INH use both as STP and as on‐demand treatment; for example, C1‐INH is not easily accessible in Brazil's National Health System despite being approved as on‐demand treatment and STP of HAE attacks, and overall access to on‐demand treatment is very limited in Brazil.^22^ The 2022 Brazilian guidelines for HAE therefore recommend C1‐INH for STP of procedures perceived to be high‐risk if C1‐INH is available, and attenuated androgens or FFP when C1‐INH is not available. For procedures perceived to be low‐risk, the 2022 Brazilian guidelines for HAE suggest observation if on‐demand medication is available and attenuated androgens when it is not.^22^ Guideline recommendations on the timing and dosing of STP medications are summarized in Table 2, although available dosing in different countries may vary based on the local labeling. Briefly, the guidelines recommend administering C1‐INH from <1 h before to 1–12 h before the procedure (the recommended timing varies in different guidelines). Androgens for STP of HAE attacks are recommended for 5–7 days before and 2–5 days after the procedure.^4^, ^9^, ^10^, ^22^

According to the guidelines, decisions to start or continue LTP should consider the efficacy and safety of the treatment, disease control, patients' QoL, and patient preference. Furthermore, the decision to start or continue LTP should be reviewed regularly as the patients' circumstances may change over time.^4^, ^9^, ^10^, ^22^ The first‐line therapies for LTP of HAE attacks include C1‐INH,^4^, ^8^, ^9^, ^10^, ^20^, ^22^ lanadelumab,^4^, ^9^, ^10^, ^20^, ^22^ and berotralstat.^4^ Antifibrinolytics and attenuated androgens are second‐line treatment options for LTP, although the 2021 WAO/EAACI guidelines on HAE management do not recommend the use of antifibrinolytics for LTP of HAE attacks.^4^, ^9^, ^10^, ^20^, ^22^

Compared with HAE Type 1/2, nC1‐INH‐HAE is a rarer form of HAE. Given the rarity of nC1‐INH‐HAE and lack of robust evidence on the efficacy and safety of HAE medications in this population, the guideline recommendations for management of patients with nC1‐INH‐HAE are scarce (Table 3). Because there are no validated biochemical tests to confirm the diagnosis of nC1‐INH‐HAE, the diagnosis is mostly based on clinical evaluation and genetic testing when available,^4^, ^9^, ^10^, ^21^ although underlying genetic cause remains unknown in up to approximately 70% of patients with nC1‐INH‐HAE.^29^, ^30^ Furthermore, the distribution of different types of mutation may vary across the global regions; for example, nC1‐INH‐HAE with mutation in the *F12* gene is reported to be exceedingly rare in the United States, but 180/300 patients with nC1‐INH‐HAE from a study in Brazil had mutation in this gene.^9^, ^31^, ^32^ Consulting physicians with expertise in angioedema conditions is highly recommended to confirm nC1‐INH‐HAE diagnosis and subsequent treatment.^9^, ^10^ No guidelines specific to pharmacologic management of patients with nC1‐INH‐HAE have been published since 2016, and some guidelines on HAE management (eg, the 2019 German guidelines on AE and the 2021 WAO/EAACI guidelines on HAE management) do not provide any recommendations for the management of nC1‐INH‐HAE.^4^, ^8^ Additionally, due to lack of data the 2019 International/Canadian HAE guidelines do not provide recommendations for LTP in patients with nC1‐INH‐HAE,^10^ and the 2020 US guidelines on HAE management include only a weak recommendation with low quality of evidence for LTP with progestin‐only medication or antifibrinolytics in patients with nC1‐INH‐HAE.^9^ Similarly, the 2022 Brazilian guidelines for HAE suggest antifibrinolytics and hormone therapy for LTP in patients with nC1‐INH‐HAE based on evidence from small open‐label studies and case series.^22^ The dearth of guideline recommendations emphasizes the unmet need for approved therapies with proven efficacy and safety in patients with nC1‐INH‐HAE.

**TABLE 3:** Summary of recent guidelines and consensus statements on HAE management in patients with nC1‐INH‐HAE.

Guideline	On‐demand treatment	STP	LTP
International guidelines			
The 2019 International/Canadian HAE guideline^10^	pdC1‐INH and icatibant are effective therapies for the acute treatment of HAE attacks in patients with nC1‐INH‐HAE.		No recommendations for LTP in nC1‐INH‐HAE could be given due to lack of data.
The 2021 WAO/EAACI guidelines on HAE management^4^	The guidelines focus on management of patients with HAE Type 1/2; management of patients with nC1‐INH‐HAE is excluded.		
National guidelines			
The 2019 German AE guideline^8^	No recommendations for management of HAE in patients with nC1‐INH‐HAE are given.		
The 2020 US HAEA Medical Advisory Board guidelines for the management of HAE^9^	Same approach as HAE Type 1/2 can be used with a caveat that on‐demand treatment be available if needed.		First step for treatment in women with nC1‐INH‐HAE is stopping exogenous estrogens. Progestin therapy and anabolic androgens are treatment options for hormonal therapy for LTP of HAE attacks in patients with nC1‐INH‐HAE. Tranexamic acid has been successfully used for LTP in patients with nC1‐INH‐HAE. Patients with nC1‐INH‐HAE with frequent attacks not manageable with other therapies could be tried on a short course of LTP with C1‐INH. A trial of lanadelumab with the same caveats as above could be available for patients with nC1‐INH‐HAE who are candidates for LTP but have failed tranexamic acid and progestins.
The 2020 Polish AE guidelines^20^	Therapy of nC1‐INH‐HAE is identical to HAE Type 1/2.		
The 2020 Brazilian guidelines for HAE^22^		As there are no published data on STP in patients with nC1‐INH‐HAE, same protocol used for STP in patients with HAE Type 1/2 should be used.	The two main therapies used for LTP in patients with nC1‐INH‐HAE are antifibrinolytics and hormone therapy. The first step of treatment of nC1‐INH‐HAE consists of suspending the use of exogenous estrogens. Other hormone therapy options include progestins or attenuated androgens. Tranexamic acid has been used for LTP in patients with nC1‐INH‐HAE with good response.
Consensus statements			
Consensus on acute treatments for HAE^24^	No recommendations for management of HAE in patients with nC1‐INH‐HAE are given.		
International consensus on diagnosis and management of pediatric patients with C1‐INH deficiency^23^	No recommendations for management of HAE in patients with nC1‐INH‐HAE are given.		
German‐speaking country consensus on therapeutic strategies in children and adolescents with HAE^26^	No recommendations for management of HAE in patients with nC1‐INH‐HAE are given.		
Argentine consensus on the diagnosis and treatment of HAE^28^	No summary is provided as the guidelines are only published in Spanish.		

Abbreviations: AE, angioedema; C1‐INH, C1 inhibitor; EAACI, European Academy of Allergy and Clinical Immunology; HAE, hereditary angioedema; HAEA, Hereditary Angioedema Association; LTP, long‐term prophylaxis; nC1‐INH‐HAE, hereditary angioedema with normal C1 inhibitor; pdC1‐INH, plasma‐derived C1 inhibitor; WAO, World Allergy Organization.

It is important to note that the new therapies for LTP in patients with HAE Type 1/2 have been incorporated in the clinical practice guidelines soon after their regulatory approvals, highlighting the need for these therapies. For example, the 2017 WAO/EAACI guidelines only had recommendations for pdC1‐INH as first‐line and attenuated androgens as second‐line options for LTP.^17^ Meanwhile, the 2021 WAO/EAACI guidelines include lanadelumab and berotralstat as well as pdC1‐INH as first‐line LTP options and retain the recommendation for attenuated androgens as a second‐line LTP option.^4^

### 2.2 Management of HAE in pregnant and breastfeeding patients

Some additional considerations apply for the pharmacologic treatment of HAE in pregnant and breastfeeding women with HAE (Table 4). PdC1‐INH is the treatment of choice for on‐demand treatment, STP, and LTP of HAE attacks.^4^, ^9^, ^10^, ^22^, ^24^ Data on the use of recombinant human C1‐INH (rhC1‐INH) in pregnancy are limited, although a case series in 14 pregnant women with HAE did not show any fetal distress, birth defects, or congenital abnormalities.^9^, ^33^ As such, the 2019 International/Canadian HAE guidelines mention the use of rhC1‐INH only in case of life‐threatening attacks during pregnancy when pdC1‐INH is unavailable or has been shown to be ineffective in an individual patient, and other current guidelines do not provide specific recommendations for the use of rhC1‐INH in pregnancy.^10^

**TABLE 4:** Summary of recent guidelines and consensus statements on HAE management in pregnant and breastfeeding patients with HAE Type 1/2.

Guideline	On‐demand treatment	STP	LTP
International guidelines			
The 2019 International/Canadian HAE guideline^10^	pdC1‐INH is the treatment of choice for acute treatment in pregnant patients. Icatibant or rhC1‐INH may be used in case of life‐threatening attacks during pregnancy when pdC1‐INH is not available or has not been efficacious for a particular patient.	STP is recommended in the case of a C‐section or intrapartum instrumentation. Attenuated androgens are not suitable in pregnancy or during breastfeeding.	pdC1‐INH is the treatment of choice when LTP is indicated in pregnant patients. Attenuated androgens should not be used during pregnancy and breastfeeding period.
The 2021 WAO/EAACI guidelines on HAE management^4^	pdC1‐INH is the preferred therapy. SDP may be used when C1‐INH is not available and FFP when SDP is not available. Ecallantide is off‐label and not recommended.	STP is recommended for interventions that come with a HAE attack risk (eg, chorionic villus sampling, amniocentesis, induced surgical abortion, or cesarean section). Preferred therapy for STP in pregnancy is pdC1‐INH.	C1‐INH is considered safe and effective prophylactic option in pregnant women when LTP is indicated. Antifibrinolytics may be considered if C1‐INH is not available, although their efficacy is not proven. Androgens are contraindicated. Lanadelumab and berotralstat are not recommended as no published experience is available.
National guidelines			
The 2019 German HAE guideline^8^	pdC1‐INH is suitable for the treatment of acute attacks during pregnancy and lactation. Icatibant has not been recommended for the treatment of acute HAE attacks to date. Use of rhC1‐INH during pregnancy and lactation is not recommended.		Androgens and tranexamic acid are contraindicated in pregnancy.
The 2020 US HAEA Medical Advisory Board guidelines on the management of HAE^9^	C1‐INH is the recommended medication for on‐demand treatment of HAE attacks. The use of icatibant in pregnancy is not endorsed until further studies are completed. pdC1‐INH or rhC1‐INH are recommended for on‐demand treatment during lactation.	STP with pdC1‐INH should be given before any procedures performed during pregnancy (eg, amniocentesis, chorionic villus sampling).	C1‐INH is the recommended medication for prophylactic HAE therapy. Anabolic androgens are specifically contraindicated. pdC1‐INH or rhC1‐INH are recommended as prophylaxis during lactation. Anabolic androgens and tranexamic acid should not be used during breastfeeding.
The 2020 Polish AE guidelines^20^			Androgens cannot be used in pregnant or breastfeeding women.
The 2022 Brazilian guidelines for HAE^22^	The therapy of choice in on‐demand treatment of HAE attacks during pregnancy, childbirth, postpartum, and breastfeeding is pdC1‐INH. FFP can be administered in cases of severe HAE attacks when pdC1‐INH is not available.	STP during pregnancy should be considered in any procedure performed, especially for procedures such as chorionic villus sampling, amniocentesis, and surgically induced abortion. Prophylaxis should be used for C‐section. The first‐choice treatment is pdC1‐INH. When STP is needed and pdC1‐INH is not available, FFP or tranexamic acid can be administered.	When LTP is necessary, the first‐line therapy is IV pdC1‐INH. When pdC1‐INH is not available, tranexamic acid may be used. Androgens are not recommended during pregnancy. Lanadelumab should not be used during pregnancy as there are currently no data available. Attenuated androgens and antifibrinolytics should be avoided during breastfeeding; however, tranexamic acid can be considered in the absence of pdC1‐INH for prophylaxis.
Consensus statements			
Consensus on acute treatments for HAE^24^	pdC1‐INH is the treatment of choice in pregnant patients with HAE. There are no safety concerns with rhC1‐INH in pregnant patients with HAE, although experience is lacking. Ecallantide and icatibant should be avoided in pregnant and breastfeeding patients with HAE due to missing evidence on safety.		

*Note*: Only guidelines available in English that discuss HAE management in pregnant and breastfeeding patients with HAE Type 1/2 were summarized.

Abbreviations: AE, angioedema; C1‐INH, C1 inhibitor; EAACI, European Academy of Allergy and Clinical Immunology; FFP, fresh frozen plasma; HAE, hereditary angioedema; HAEA, Hereditary Angioedema Association; LTP, long‐term prophylaxis; pdC1‐INH, plasma‐derived C1 inhibitor; rhC1‐INH, recombinant human C1 inhibitor; SDP, solvent detergent–treated plasma; STP, short‐term prophylaxis; WAO, World Allergy Organization.

STP is recommended for medical procedures related to pregnancy, such as chorionic villus sampling and amniocentesis.^4^, ^9^, ^10^, ^22^ STP is also recommended when undergoing cesarean section; however, it is not usually required for uncomplicated vaginal delivery, with a caveat that on‐demand treatment should be readily available.^4^, ^10^, ^22^ Due to lack of data in pregnant patients with HAE, the newer HAE‐specific therapies such as icatibant and ecallantide for on‐demand treatment of HAE attacks or lanadelumab and berotralstat for the prevention of HAE attacks are not recommended in this population.^4^, ^22^, ^24^ No guidelines provide recommendations for managing LTP in pregnant patients with HAE who have been receiving lanadelumab or berotralstat for LTP prior to pregnancy. Some of the treatment options that are second‐line for the patients with HAE overall, such as solvent detergent–treated plasma (SDP) or FFP may be considered for on‐demand treatment and tranexamic acid may be considered for prophylaxis in pregnant patients with HAE when C1‐INH is not available.^4^, ^22^ All recent guidelines include specific recommendation against the use of androgens in pregnant and breastfeeding patients.^4^, ^8^, ^9^, ^10^, ^20^, ^22^

### 2.3 Management of HAE in pediatric patients

Management of HAE in pediatric patients (aged <18 years) also requires additional considerations (Table 5). Country and patients' age must be taken into the account as well since the age groups for which the treatment options recommended by international guidelines are approved may vary per country.^10^, ^22^, ^26^ The guideline‐recommended treatment options for on‐demand treatment of HAE attacks in pediatric patients include C1‐INH^4^, ^8^, ^9^, ^10^, ^20^, ^22^, ^23^, ^24^, ^26^ and icatibant,^4^, ^8^, ^10^, ^22^, ^24^, ^26^ with ecallantide also available for adolescent patients in some countries, including the United States and several countries in Latin America.^4^, ^9^, ^10^, ^23^, ^24^ Where these therapies are not available, FFP or SDP may be used, with SDP preferred over FFP.^4^, ^22^, ^23^ STP in pediatric patients with HAE follows similar recommendations to STP in adults: pdC1‐INH and attenuated androgens are the treatment options, and plasma may be considered if these therapies are not available.^4^, ^9^, ^22^, ^23^, ^26^ The 2017 international consensus statement on diagnosis and management of pediatric HAE includes tranexamic acid alongside attenuated androgens as an option for STP when on‐demand treatment is not available for planned procedures, in line with recommendations in older consensus statements (eg, the 2005 consensus statement on C1‐INH deficiency).^23^, ^34^ pdC1‐INH is the recommended therapy for LTP in pediatric patients with HAE, although additional first‐line options may be available to adolescent patients aged >12 years^4^, ^9^, ^10^, ^22^, ^26^ A consensus statement on management of HAE in pediatric patients from German‐speaking countries (Germany, Austria, and Switzerland) as well as the 2022 Brazilian guidelines for HAE also recommend lanadelumab for LTP in patients with HAE aged >12 years^22^, ^26^ Androgens are not recommended in pediatric patients, but they may be considered in adolescent patients after Tanner stage 5.^4^, ^9^, ^10^, ^20^, ^22^, ^23^, ^26^ There is a lack of consensus for the use of antifibrinolytics in pediatric patients with HAE for the LTP of HAE attacks. The 2019 international/Canadian guidelines and the 2019 German‐speaking country consensus do not recommend antifibrinolytics for LTP in pediatric patients with HAE due to lack of proven efficacy while the 2021 WAO/EAACI and the 2022 Brazilian guidelines list antifibrinolytics as second‐line LTP options for pediatric patients with HAE.^4^, ^10^, ^22^, ^26^ A consensus statement on diagnosis and management of pediatric HAE published in 2017 is the only guidance document to recommend tranexamic acid as the first‐line LTP option in pediatric patients with HAE.^23^

**TABLE 5:** Summary of recent guidelines and consensus statements on HAE management in pediatric patients with HAE Type 1/2.

Guideline	On‐demand treatment	STP	LTP	
International guidelines			
The 2019 International/Canadian HAE guideline^10^	IV pdC1‐INH, icatibant and IV rhC1‐INH are effective therapies for the acute treatment of HAE attacks in pediatric patients. Ecallantide is an effective therapy for acute treatment of HAE attacks in adolescent patients.		pdC1‐INH is the treatment of choice when LTP is indicated in pediatric patients. Attenuated androgens should not be used in pediatric patients. Androgens are contraindicated as LTP in pediatric patients before Tanner stage 5 but may be considered once patients have completed puberty. Antifibrinolytics cannot be recommended for LTP in pediatric patients due to lack of evidence.
The 2021 WAO/EAACI guidelines on HAE management^4^	C1‐INH or icatibant are recommended for the treatment of HAE attacks in patients <12 years of age. When C1‐INH and icatibant are not available, SDP and FFP are second‐line therapies, with SDP preferred over FFP. Ecallantide is licensed for adolescents in the United States.	pdC1‐INH is the first‐line STP option in pediatric patients with HAE Type 1/2. Attenuated androgens should only be used as a second‐line treatment option when C1‐INH is not available. On‐demand therapy should be available with either STP option.	Preferred therapy for LTP in patients <12 years of age is pdC1‐INH. When C1‐INH is not available, antifibrinolytics are preferred to androgens, although efficacy data are lacking. Androgens are not recommended for LTP in children and adolescents prior to Tanner stage 5.
National guidelines			
The 2019 German HAE guideline^8^	pdC1‐INH, icatibant, and rhC1‐INH are approved for the treatment of acute attacks in children.		
The 2020 US HAEA Medical Advisory Board guidelines on the management of HAE^9^	pdC1‐INH is the treatment of choice for on‐demand treatment of pediatric patients. Ecallantide is approved for children >12 years of age.	Indications for STP in pediatric patients follow the same guidelines as in adults.	The preferred LTP in pediatric patients is pdC1‐INH. SC C1‐INH and lanadelumab are approved for children >12 years of age. Anabolic androgens are not recommended for pediatric patients.
The 2020 Polish AE guidelines^20^			Androgens cannot be used in children.
The 2022 Brazilian guidelines for HAE^22^	IV pd‐C1‐INH and icatibant (in patients aged >2 years) are the first‐line on‐demand treatments. Plasma is a second‐line STP treatment. For treatment of HAE attacks in patients aged <12 years, icatibant may be used in patients aged >2 years; IV pdC1‐INH and FFP can be used without age restrictions.	IV pdC1‐INH is the first‐line STP treatment. Attenuated androgens and plasma are second‐line STP treatments. For STP, the same pharmacotherapy strategies as used in adults are recommended. Although attenuated androgens are not indicated for LTP in children before puberty, they can be used for STP before risky procedures.	IV pdC1‐INH, SC pdC1‐INH (in patients aged >8 years), and lanadelumab (in patients aged >12 years) are first‐line LTP options. Tranexamic acid and attenuated androgens (after puberty) are second‐line treatment options. Tranexamic acid is available for LTP in patients aged <8 years, patients between 8 and 12 years can receive SC pdC1‐INH, and patients aged ≥12 years can receive same LTP as adult patients, considering attenuated androgens in pediatric patients with Tanner stage 5. Danazol is contraindicated in children (pediatric patients at Tanner stage 1–4).
Consensus statements			
Consensus on acute treatments for HAE^24^	pdC1‐INH is the treatment of choice in preadolescent children with HAE. There are no safety concerns with rhC1‐INH in preadolescent children with HAE, although experience is lacking. Ecallantide and icatibant may have a role in the treatment of children with HAE.		
International consensus on diagnosis and management of pediatric patients with C1‐INH deficiency^23^	pdC1‐INH and rhC1‐INH are licensed for acute treatment in pediatric patients, as well as ecallantide in patients aged ≥12 years.	For most minor interventions, on‐demand treatment is preferred over STP. STP with pdC1‐INH is recommended for interventions that involve airway manipulation or may induce swelling. If on‐demand treatment medications are not available, STP with attenuated androgens or antifibrinolytics is recommended. SDP may be used for STP or on‐demand treatment after procedure if other options are not available.	Tranexamic acid is the LTP of choice in pediatric patients with HAE. When antifibrinolytics fail to achieve the effect, are contraindicated or not tolerated, pdC1‐INH is recommended. Attenuated androgens are not considered in pediatric patients before Tanner stage 5, but can be used after. pdC1‐INH may be the safest LTP option and is recommended over attenuated androgens.
German‐speaking country consensus on therapeutic strategies in children and adolescents with HAE^26^	IV C1‐INH is recommended in pediatric patients with HAE aged <2 years. IV C1‐INH or icatibant are recommended for pediatric patients with HAE aged ≥2 years.	IV C1‐INH is recommended for STP in pediatric patients with HAE.	IV C1‐INH is recommended for LTP in pediatric patients with HAE aged 6 to <12 years. IV C1‐INH, SC C1‐INH and lanadelumab are recommended for LTP in pediatric patients with HAE aged ≥12 years. Androgen use is considered contraindicated in pediatric patients with HAE. Tranexamic acid is not recommended despite existing approval.

*Note*: Only guidelines available in English that discuss HAE management in pediatric patients with HAE Type 1/2 were summarized.

Abbreviations: AE, angioedema; C1‐INH, C1 inhibitor; EAACI, European Academy of Allergy and Clinical Immunology; FFP, fresh frozen plasma; HAE, hereditary angioedema; HAEA, Hereditary Angioedema Association; IV, intravenous; LTP, long‐term prophylaxis; pdC1‐INH, plasma‐derived C1 inhibitor; rhC1‐INH, recombinant human C1 inhibitor; SC, subcutaneous; SDP, solvent detergent–treated plasma; STP, short‐term prophylaxis; WAO, World Allergy Organization.

### 2.4 HAE treatment patterns in specific countries

**FIGURE 2:**
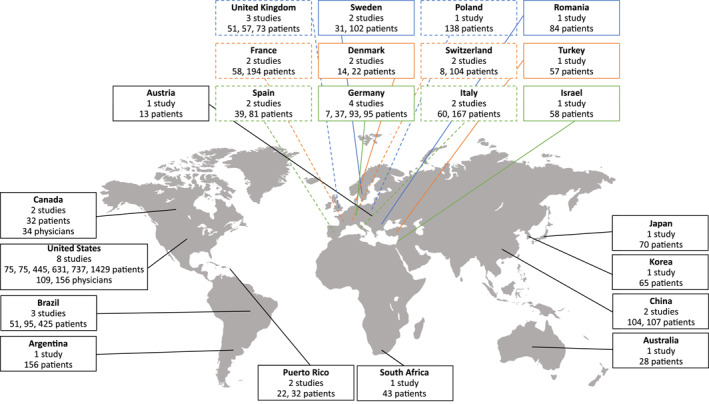
Summary of studies from 2016 to 2021 reporting on HAE treatment patterns in specific countries.

The guideline recommendations for newer HAE‐specific therapies are consistent; however, these therapies may be unavailable or difficult to access in some countries due to lack of local approvals or prohibitively high costs. This disparity in HAE treatment availability between countries has been recently summarized by Jindal et al.^35^ Furthermore, the availability of published data on HAE treatment patterns also varies among countries (Figure 2). For example, six studies on the treatments received by patients with HAE and two studies on HAE treatments prescribed by the physicians in the United States included data from up to 1429 patients with HAE and up to 156 physicians treating HAE per study.^36^, ^37^, ^38^, ^39^, ^40^, ^41^, ^42^, ^43^ In contrast, we have found only one study from the continent of Africa reporting on HAE treatment patterns; this study included 43 patients with HAE from South Africa.^44^ The low number of patients reported in some country‐specific studies suggests that many patients with HAE remain undiagnosed in these countries, taking into the account the estimated HAE prevalence rates and the populations of these countries. For example, the estimated proportion of countries' total population of patients with HAE enrolled in the Hereditary Angioedema Global Registry ranged from 14.4% (Greece) to 91.2% (Hungary).^45^ This is perhaps unsurprising as long delays between the onset of HAE symptoms and HAE diagnosis are still reported by multiple recent studies, each from a different country: a mean of 7.75 years in Korea,^46^ a median of 10 years in Sweden,^47^ a mean of 14 years in Switzerland,^48^ a mean of 15.6 years in Romania,^49^ and 17 years in Turkey.^50^

The available information on country‐specific HAE treatment patterns since 2016 is summarized in Table 6. In most of the countries with available data, attenuated androgens and antifibrinolytics remain widely used for HAE prophylaxis, although these medications are no longer recommended as first‐line treatment options for LTP in the international guidelines. It is important to note that some of these studies report data collected before the newest prophylactic therapies (lanadelumab and berotralstat) were approved in the United States and the EU, whereas others report data from countries where attenuated androgens and/or tranexamic acid remain the only medications approved for LTP of HAE attacks. Attenuated androgens may also have a role in management of HAE in specific patients even when cost and access to first‐line therapies is not an issue, for example, in patients who have been historically managed with androgens and obtained benefit from their use.^10^ In these cases, lowest effective dose is recommended to avoid side effects, and maximum daily dose (200 mg for danazol, as per expert consensus) should not be exceeded.^4^, ^9^, ^10^, ^22^

**TABLE 6:** Summary of recent papers reporting on country‐specific HAE treatment patterns.

Reference	Population	On‐demand treatment		STP	LTP
North America					
United States					
Riedl et al. (2018)^36^	Database analysis of 631 patients with HAE and ≥1 insurance claim for an HAE‐specific medication for the period 1 January 2006 to 31 December 2014.	31.2% of patients exclusively had claims for ecallantide or icatibant without concomitant use of IV C1‐INH.			24.6% of patients (155/631) had 306 episodes of prophylaxis with IV C1‐INH, with a mean duration of 339 days. On‐demand medication was used during 53% (163/306) prophylactic episodes.
Banerji et al. (2020)^39^	Survey in 445 patients with HAE Type 1/2 who completed the survey between 17 March 2017 and 28 April 2017.			29.9% of patients reported that they had taken or were currently taking STP.	68.5% of patients reported ongoing or past LTP. 60.7% patients were using C1‐INH for short‐ or long‐term prophylaxis, 27.2% were using androgens (danazol, stanozolol, oxandrolone, and methyltestosterone); 0.9% of patients reported LTP with aminocaproic acid.
Tachdjian et al. (2020)^42^	Database analysis of 1429 patients identified as likely to have HAE during the study period of 1 January 2006 through 30 September 2015.	The proportion of patients who received C1‐INH (for on‐demand treatment or prophylaxis), ecallantide, or icatibant was 81.8% in 2015, increased from 0.9% in 2006. The proportion of patients who received androgens was 24.9% in 2015, decreased from 91.5% in 2006. The proportion of patients treated with FFP, tranexamic acid, or epsilon‐aminocaproic acid was 5.2% in 2015, decreased from 15.2% in 2008.			
Geba et al. (2021)^40^	Survey of 75 patients with HAE Type 1/2 who completed the survey between 5 November and 3 December 2018.				64% of patients used medication for LTP; 29% were using lanadelumab, 33% were using C1‐INH, and 8% were using androgens for LTP of HAE attacks within 12 months of completing the survey.
Radojicic et al. (2021)^41^	Survey of 75 patients with HAE Type 1/2 who completed the survey between 20 May and 10 June 2020.				53.3% of patients were using lanadelumab and 25.3% of patients were using SC C1‐INH for their LTP. 8% of patients reported using IV C1‐INH and 4% reported using androgens for their LTP.
Castaldo et al. (2021)^43^	Survey of 737 patients with HAE Type 1/2 who completed an online survey from 9 to 28 July 2018.	275 patients (37.3%) used on‐demand treatment only.			149 patients (20.2%) used SC C1‐INH or lanadelumab, 138 patients (18.7%) used C1‐INH, 84 patients (11.4%) used androgens, 14 patients (1.9%) used rhC1‐INH, 63 patients (8.5%) used a combination of agents, and 14 (1.9%) patients used other medications for LTP.
Riedl et al. (2021a)^37^	Survey of 156 physicians treating HAE who filled the questionnaire during June and July 2019.	Icatibant was the most frequently prescribed agent for acute treatment of attacks (71.7%), followed by pdC1‐INH (52.9%) and rhC1‐INH (31.9%). Ecallantide was prescribed by 29.0% of physicians, FFP by 10.1% of physicians; 1.5% of physicians reported prescribing no acute HAE treatment.		87.5% of physicians reported prescribing STP in high‐risk situations, and 72.8% reported that C1‐INH remains the treatment of choice for STP.	63.3% physicians reported that >40% of their patients were receiving LTP. 60.0% of physicians reported that they most frequently prescribe C1‐INH for LTP; 21.4% reported most frequently prescribing lanadelumab and 6.4% reported most frequently prescribing danazol. 59.9% of physicians reported not prescribing any attenuated androgens.
Riedl et al. (2021b)^38^	Survey of 109 physicians treating HAE who completed the survey between 19 May and 14 June 2020.				63% of physicians reported currently prescribing SC C1‐INH, 41% currently prescribing IV C1‐INH, 52% currently prescribing lanadelumab, 38% currently prescribing androgens, and 11% currently prescribing antifibrinolytics.
Canada					
Fu et al. (2018)^64^	Survey in 34 physicians who were treating patients with HAE Type 1/2, with some physicians also treating patients with nC1‐INH‐HAE, conducted January–February 2017.	Prescription of C1‐INH for on‐demand treatment was reported by 88.2% of physicians, prescription of icatibant by 79.4% of physicians, and prescription of tranexamic acid and danazol was reported by 5.9% of physicians each. 2 physicians reported exclusively prescribing icatibant and 4 physicians reported exclusively prescribing C1‐INH for on‐demand treatment of attacks.		27/34 physicians (79.4%) reported prescribing STP for their HAE patients. The agents prescribed included C1‐INH (76.5%), danazol (11.8%), and tranexamic acid (2.9%).^a^	Physicians reported that an average of 53.9% of their patients with HAE Type 1/2 were on LTP. 93.5% of physicians reported prescribing C1‐INH, 41.9% reported prescribing danazol, and 19.4% reported prescribing tranexamic acid for LTP.
Mendivil et al. (2021)^62^	32 patients from Canada who participated in an international survey from July to October 2018.			21/32 patients reported having used STP; 2 different formulations of C1‐INH were reported by 2 and 18 patients, tranexamic acid by 2 patients, and danazol by 1 patient.	23/32 patients reporting having used LTP; 2 different formulations of C1‐INH were reported in 4 and 16 patients, tranexamic acid in 3 patients, danazol in 1 patient, and other medications in 3 patients.
Europe					
Austria					
Mendivil et al. (2021)^62^	13 patients from Austria who participated in an international survey from July to October 2018.			8/13 patients reported STP, all with C1‐INH.	2/13 patients reported LTP, 1 with C1‐INH and 1 with other medications.
Denmark					
Aabom et al. (2017a)^58^	22 children with HAE Type 1/2 from Denmark, 14 of whom were symptomatic, who were observed from January 2013 to October 2016.	On‐demand medications used at any time, including prior to observation period, were tranexamic acid (9/14 symptomatic patients), C1‐INH (12/14 symptomatic patients), and icatibant (1/14 symptomatic patients). Home therapy with C1‐INH was established in 9 cases: self‐administration in 6 patients and administration by parents in 3 cases.		Medications for STP used at any time, including prior to observation period, included tranexamic acid (4/14 symptomatic patients) and C1‐INH (5/14 symptomatic patients).	Medications used for LTP used at any time, including prior to observation period, included tranexamic acid (4/14 symptomatic patients) and C1‐INH (1/14 symptomatic patients; the use was off‐label at the time this study was published).
Aabom et al. (2017b)^85^	14 pediatric patients from Denmark who participated in an observational study from May 2013 to August 2014.	At or from first assessment, 7 patients reported on‐demand therapy with C1‐INH and 2 with tranexamic acid. At or from second assessment, 8 patients reported on‐demand therapy with C1‐INH, 2 with tranexamic acid, and 1 with bradykinin receptor antagonist.		At or from second assessment, 1 patient reported STP with C1‐INH.	At or from first and second assessments, 1 patient reported LTP with tranexamic acid.
France					
Caballero et al. (2017)^59^	194 patients from France who were enrolled in the Icatibant Outcome Survey between July 2009 and April 2015.				LTP was reported in 34.3% of patients. Recorded LTP medications were androgens (54.9%), tranexamic acid (39.4%), C1‐INH (2.1%), and combination of multiple therapies (3.5%).
Mendivil et al. (2021)^62^	58 patients from France who participated in an international survey from July to October 2018.			30/58 patients reported using STP; 2 different formulations of C1‐INH were reported in 4 and 11 patients, tranexamic acid in 1 patient, and danazol in 13 patients.	35/58 patients reported using LTP; 2 different formulations of C1‐INH were reported in 7 and 2 patients, tranexamic acid in 2 patients, danazol in 15 patients, and other medications in 15 patients.
Germany					
Caballero et al. (2017)^59^	95 patients from Germany and Austria who were enrolled in the Icatibant Outcome Survey between July 2009 and April 2015.				LTP was reported in 11.8% of patients. Recorded LTP medications were androgens (53.3%) and C1‐INH (46.7%).
Maurer et al. (2019)^65^	93 patients from Germany who were enrolled in the Icatibant Outcome Survey between July 2009 and January 2017.		Ongoing LTP or STP at entry to the Icatibant Outcome Survey or during the follow‐up period with C1‐INH was reported in 65.0% of patients and with danazol in 40.0% of patients.		
Zarnowski et al. (2021)^89^	37 patients who were treated in Angioedema Center of Reference and Excellence in the Department of Dermatology, Venereology, and Allergology at the University of Leipzig.	54.1% of patients (20/37) were treated with on‐demand treatment only and 37.8% of patients (14/37) were treated with on‐demand treatment in addition to prophylaxis.			45.9% of patients (17/37) were receiving LTP; 3 were treated with danazol, 2 were treated with lanadelumab, and the majority were treated with C1‐INH.
Mendivil et al. (2021)^62^	Seven patients from Germany who participated in an international survey from July to October 2018.			6/7 patients reported using STP; 2 different formulations of C1‐INH were reported in 1 and 5 patients.	1/7 patients reported using LTP with C1‐INH.
Italy					
Caballero et al. (2017)^59^	60 patients from Italy who were enrolled in the Icatibant Outcome Survey between July 2009 and April 2015.				LTP was reported in 34.2% of patients. Recorded medications for LTP were androgens (64.4%), tranexamic acid (11.1%), C1‐INH (17.8%), and androgens/tranexamic acid (6.7%).
Federici et al. (2018)^56^	167 patients with HAE who were treated at Milan angioedema center between 1 January and 31 December 2014.	704/1508 attacks were treated with pdC1‐INH, 486/1508 attacks were treated with icatibant, and no treatment was administered for 318/1508 attacks.			29/133 patients who reported HAE attacks were receiving LTP, with 15 patients receiving danazol, 11 patients receiving stanozolol, and 3 patients receiving tranexamic acid.
Poland					
Piotrowicz‐Wojcik (2021)^57^	138 adult patients with HAE Type 1/2 who were interviewed by their treating physicians between January 2018 and June 2019.	103/118 symptomatic patients used on‐demand treatment for HAE attacks in prior 6 months: 81 used pdC1‐INH, 55 used icatibant, and 10 used rhC1‐INH. 61.4% of attacks were treated with pdC1‐INH, 36.7% with icatibant, and 1.9% with rhC1‐INH. 6 patients did not have any on‐demand treatment drugs at home; others had pdC1‐INH, icatibant, rhC1‐INH, or ≥1 drug at home. 116 patients carried the on‐demand medication while traveling. 74 patients reported self‐administration of on‐demand treatment.		STP use in the last 6 months was reported by 31 patients for 55 occasions. In 4 cases, patients administered icatibant themselves despite the recommendations for STP. pdC1‐INH was used in all other cases.	68/138 patients reported history of LTP. 24/68 reported having used danazol, 25/68 reported having used tranexamic acid, and 19/68 reported using both danazol and tranexamic acid, but at different times. 23 patients used LTP in the last 6 months; 16/23 had used danazol and 7/23 had used tranexamic acid.
Romania					
Gabos et al (2020)^49^	84 patients with HAE Type 1/2 who were interviewed from July to October 2018.	pdC1‐INH was used in 11.9% of patients and rhC1‐INH in 15.5% for the treatment of life‐threatening attacks involving the airway or gastrointestinal tract in the emergency department in the previous 12 months 14 (16.7%) patients reported the use of FFP for management of acute attacks in the previous 12 months 51.2% patients reported home treatment with icatibant.			No patients were receiving LTP with C1‐INH. 23/84 (27.3%) patients were receiving LTP with androgens (17/23 danazol, 6/23 other androgens).
Spain					
Caballero et al. (2017)^59^	81 patients from Spain who were enrolled in the Icatibant Outcome Survey between July 2009 and April 2015.				LTP was reported in 41.7% of patients. Recorded LTP medications were androgens (35.6%), tranexamic acid (53.4%), C1‐INH (9.2%), and androgens/tranexamic acid (1.8%).
Mendivil et al. (2021)^62^	39 patients from Spain who participated in an international survey from July to Oct 2018.			30/39 patients reported using STP; 2 different formulations of C1‐INH were reported in 5 and 19 patients, tranexamic acid in 1 patient, danazol in 8 patients, and stanozolol in 4 patients.	28/39 patients reported LTP; 2 different formulations of C1‐INH in 1 and 7 patients, tranexamic acid in 2 patients, danazol in 11 patients, stanozolol in 10 patients, and other medications in 1 patient.
Sweden					
Nygren et al. (2016)^60^	31 patients <18 years of age from Swedish national registry of patients with HAE (Sweha‐Reg) who answered a questionnaire between 2007 and 2011.	19/29 patients (66%) reported having access to pdC1‐INH at home, with 5 patients having availability to receive IV treatment at home by a trained relative. 9 patients had used IV C1‐INH, and 5 had used tranexamic acid for acute treatment, but none had received FFP.			9 patients were using LTP: antifibrinolytics in 4, combination of antifibrinolytics and C1‐INH in 1, and oxandrolone in 4.
Nordenfelt et al. (2016)^47^	Adult patients with HAE Type 1/2 registered in Swedish national registry of patients with HAE (Sweha‐Reg) between 2007 and 2011, who answered the written questionnaire (*n* = 102) and/or participated in telephone interview (*n* = 99).	27% of patients have received pdC1‐INH for on‐demand treatment of attacks. 16% reported having used tranexamic acid, 8% FFP, and 2% androgens for on‐demand treatment of attacks.			88 patients had history of LTP at any point in their lives; 49% reported previous use of antifibrinolytics, 43% of androgens and 8% of pdC1‐INH. 22/88 (25.0%) of patients were using androgens at the time of answering the questionnaire.
Switzerland					
Steiner et al. (2016)^48^	104 patients with HAE Type 1/2 (57 women and 47 men) who were surveyed in 2013 about their medication use retrospectively for year 2012.	57/104 patients (32/57 [56%] women and 25/37 [53%] men) used pdC1‐INH; 12/104 patients (9/57 [16%] women and 3/47 [6%] men) used icatibant for on‐demand treatment. 38 patients (23 women and 15 men) reported home treatment with C1‐INH.			10/104 patients (6/57 [10%] women and 4/47 [8%] men) used tranexamic acid, and 26/104 patients (13/57 [23%] women and 13/47 [27%] men) used danazol for LTP.
Mendivil et al. (2021)^62^	8 patients from Switzerland who participated in an international survey from July to October 2018.			4/8 patients reported using STP; a single formulation of C1‐INH was reported in all patients.	2/8 patients reported using LTP; C1‐INH was reported in 1 and other medications in 1 patient.
United Kingdom					
Caballero et al. (2017)^59^	51 patients from the United Kingdom who were enrolled in the Icatibant Outcome Survey between July 2009 and April 2015.				LTP was reported in 55.6% of patients. Recorded medications for LTP were androgens (69.9%), tranexamic acid (4.8%), C1‐INH (5.6%), and combination of multiple therapies (19.7%).
Longhurst et al. (2018)^61^	73 patients from the United Kingdom with HAE Type 1/2 who were enrolled in the Icatibant Outcome Survey between July 2009 and July 2016.				55/73 patients (75.3%) were receiving ongoing LTP. Attenuated androgens (danazol, stanozolol, and oxandrolone) accounted for 59.9% of LTP usage. 4/55 patients (7.3%) used C1‐INH and 13/55 patients (23.6%) used tranexamic acid for their LTP.
Mendivil et al. (2021)^62^	57 patients from the United Kingdom who participated in an international survey from July to October 2018.			39/57 patients reported using STP; 2 different formulations of C1‐INH were reported in 7 and 28 patients, tranexamic acid in 3 patients, and danazol in 3 patients.	38/57 patients reported LTP; 2 different formulations of C1‐INH in 8 and 13 patients, tranexamic acid in 12 patients, danazol in 7 patients, stanozolol and oxandrolone in 1 patient each, and other medications in 4 patients.
South America					
Argentina					
Malbran et al. (2017)^96^	156 patients with HAE from the Argentina HAE patient association who filled online questionnaire in 2016.	C1‐INH was accessible for 86/156 patients, icatibant for 10/156 patients, and both for 22/156 patients. 38/156 patients had no access to both C1‐INH and icatibant. 32 patients reported self‐administration and 19 reported administration by family member.			
Brazil					
Fragnan et al. (2018)^52^	51 patients with HAE Type 1/2 who were attending the specialized outpatient clinic at Faculdade de Medicina de ABC between December 2009 and November 2017.	25/40 (67.5%) patients who were receiving LTP had breakthrough attacks. 5 patients did not have access to on‐demand treatment and had the dose of tranexamic acid, oxandrolone or danazol increased during attacks. 17 patients reported current use of icatibant, 1 was using C1‐INH, and 3 FFP for on‐demand treatment of attacks.			40/46 symptomatic patients underwent any LTP. 23/40 reported the use of a single agent (tranexamic acid and oxandrolone: 8/23 each, danazol: 6/23, epsilon‐aminocaproic acid: 1/23). 17/40 patients reported the use of several medications for LTP.
Veronez et al. (2021)^31^	425 patients with HAE (125 with HAE Type 1/2, 180 with nC1‐INH‐HAE with mutation in the *F12* gene, 120 with nC1‐INH‐HAE with unknown mutation) who were analyzed retrospectively.	Attenuated androgens and tranexamic acid were used for on‐demand treatment of attacks in 20% of patients with HAE Type 1/2. 17 patients with HAE Type 1/2, 8 patients with nC1‐INH‐HAE with *F12* mutation, and 16 patients with nC1‐INH‐HAE with unknown mutation treated acute attacks with C1‐INH and/or icatibant.			LTP with attenuated androgens and tranexamic acid was administered in 52% and 20% of symptomatic patients with HAE Type 1/2, respectively; 7% and 18% of patients with nC1‐INH‐HAE with mutation in *F12* gene, respectively; 14% and 28% of patients with nC1‐INH‐HAE with unknown mutation, respectively. A total of 5 patients from these groups used C1‐INH for LTP.
Araújo‐Simões et al. (2021)^51^	95 patients <18 years of age with HAE from Brazil with the data from the first clinical evaluation until Dec 2018 who were analyzed retrospectively.	On‐demand treatment with FFP was reported in 20.0% (16/80) symptomatic patients, C1‐INH in 13.8% (11/80), and icatibant in 8.8% (7/80). 17.5% (14/80) of symptomatic patients reported using other medications for the treatment of HAE attacks.		11.3% (9/80) symptomatic patients had used STP; tranexamic acid was used in 6/80 patients, and danazol in 3/80 patients >12 years of age.	65% (52/80) of symptomatic patients used LTP; tranexamic acid in 39/80, danazol in 7/80, and oxandrolone in 6/80.
Puerto Rico					
Arce‐Ayala et al. (2019)^66^	32 patients with HAE from Puerto Rico surveyed from 2015 to 2016.	81.3% (26/32) of patients reported using on‐demand treatment; 21 patients reported using icatibant, 2 patients reported using ecallantide, 1 patient reported using C1‐INH, and 2 patients reported using several different medications for on‐demand treatment.			58.1% (18/31) of patients reported using prophylaxis. In those using prophylaxis, 11 patients used C1‐INH, 3 patients used danazol, and 2 patients used several different treatments for prophylaxis.
Rosado‐Quiñones et al. (2019)^97^	22 patients with HAE (18 Type 1, 4 Type 2) from Puerto Rico who were evaluated between 2013 and 2016.	17 patients (77.3%) received icatibant and/or ecallantide for on‐demand treatment of HAE attacks.			10 patients (45.5%) received C1‐INH for prophylaxis.
Asia					
China					
Liu et al. (2019)^53^	104 patients with HAE from China.				74 patients were currently receiving LTP with danazol, 15 patients had discontinued danazol prophylaxis due to presence of or worry about side effects, and 17 patients had never initiated LTP with danazol.
Liu et al. (2020)^54^	107 patients with HAE Type 1/2 from China who participated in an internet‐based survey.	10 patients reported 18 cases of virally inactivated FFP transfusions: 16 for on‐demand treatment of attacks, and 2 as prophylaxis after a severe trauma.			83.2% (89/107) of patients reported having used danazol for LTP at any time. 74/107 (69.2%) patients were using danazol at the time the survey was conducted, and 15 discontinued. 18 patients had never used danazol. 3.7% (4/107) patients reported current or past use of tranexamic acid. 1 patient discontinued tranexamic acid.
Israel					
Toubi et al. (2018)^55^	58 patients from Israel with HAE Type 1/2 who were enrolled in the Icatibant Outcome Survey up to July 2016.				14/58 (24.1%) patients reported the use of LTP, all of them with danazol.
Japan					
Iwamoto et al. (2021)^63^	70 patients with HAE from Japan who filled a questionnaire between July and November 2016.	pdC1‐INH was prescribed for the treatment of acute attacks in 51/62 patients (82.3%) who experienced HAE attacks. Other medications for acute treatment included oral tranexamic acid in 14/62 (22.6%) patients, tranexamic acid injections in 12/62 (19.4%) patients, and danazol in 4/62 (6.5%) patients.	42/70 patients used prophylaxis regularly, 8/70 took prophylactic medication only when they felt an attack was likely, and 13/70 had never used LTP. 38/49 patients (77.6%) used tranexamic acid for LTP, 10/49 (20.4%) used danazol, and 1/49 (2.0%) used C1‐INH.		
Korea					
Jung et al. (2018)^46^	65 patients with HAE from Korea who were diagnosed by 2016.				44/65 patients (67.7%) used danazol for LTP; 6 of them used tranexamic acid together with danazol for their LTP.
Turkey					
Demir et al. (2019)^50^	57 patients >17 years of age with HAE with a history of C1‐INH use for the treatment of HAE attacks.	In addition to C1‐INH treatment, patients reported using increased dose of danazol (7/57 patients [12.3%]), icatibant (17/57 patients [29.8%]), and FFP (2/57 patients [3.5%]) for the treatment of HAE attacks.			40/57 patients were receiving LTP; 39 with danazol and 1 with tranexamic acid.
Africa					
South Africa					
Coovadia et al. (2018)^44^	43 patients with HAE type 1 from Western Cape in South Africa who were analyzed retrospectively.	11/43 (25.6%) patients used FFP for the acute treatment of attacks, 4/43 (9.3%) used icatibant, and 1/43 patients received C1‐INH for acute treatment.		4/43 patients (9.3%) used C1‐INH as STP.	21/43 patients (48.8%) used danazol for LTP.
Australia					
Australia					
Mendivil et al. (2021)^62^	28 patients from Australia who participated in an international survey from July to October 2018.			18/28 patients reported STP; 2 different formulations of C1‐INH for were reported in 1 and 13 patients, tranexamic acid in 1 patient, danazol in 6 patients, and other medications in 2 patients.	22/28 patients reported using LTP. A single formulation of C1‐INH was used in 10 patients, tranexamic acid in 8 patients, and danazol in 9 patients.

Abbreviatons: C1‐INH, C1 inhibitor; FFP, fresh frozen plasma; HAE, hereditary angioedema; IV, intravenous; LTP, long‐term prophylaxis; nC1‐INH‐HAE, hereditary angioedema with normal C1‐INH; pdC1‐INH, plasma‐derived C1 inhibitor; rhC1‐INH, recombinant human C1 inhibitor; SC, subcutaneous; STP, short‐term prophylaxis.

^a^ Due to a programming error that was later fixed, 15 respondents could only select one response and 12 respondents could select all available responses.

In line with lack of approvals and/or limited availability for HAE‐specific medications, studies from multiple countries only reported the use of attenuated androgens and tranexamic acid for LTP in HAE. These included studies from Brazil,^51^, ^52^ China,^53^, ^54^ Israel,^55^ Italy,^56^ Korea,^46^ Poland,^57^ Romania,^49^ South Africa,^44^ Turkey,^50^ and Switzerland.^48^ Interestingly, 6/12 of these studies reported that >50% of the investigated patients were receiving LTP, suggesting that even second‐line LTP options are considered useful in real‐world practice when the first‐line options are not available or affordable. The studies from China reported that some patients did not start or discontinued LTP with attenuated androgens due to side effects or worry about side effects,^53^, ^54^ indicating that patients who may benefit from LTP are not receiving it likely due to unavailability of treatment options considered suitable by the patients or their physicians. This further highlights the unmet need for newer HAE‐specific medications in countries where they are not yet available.

Many of the countries that reported the use of C1‐INH for LTP of HAE attacks nevertheless mainly used second‐line therapies such as androgens and/or antifibrinolytics.^47^, ^56^, ^58^, ^59^, ^60^, ^61^ These countries included Denmark (C1‐INH use for LTP reported in 7.1% of symptomatic patients),^58^ France (C1‐INH use for LTP reported in up to 15.5% of patients),^62^ Japan (C1‐INH use reported in 2.0% patients),^63^ Spain (C1‐INH use for LTP reported in up to 20.5% of patients),^62^ and Sweden (history of C1‐INH for LTP reported in up to 8% of patients).^47^ The HAE Global Registry data collected from five European countries (France, Greece, Hungary, Italy, and Romania) between 1 January 2018 and 31 August 2020 showed that out of 389 patients taking prophylactic medication, 240 received attenuated androgens, further highlighting widespread use of androgens in Europe despite other approved treatment options being available.^45^ The same registry also reported LTP with tranexamic acid in 44 patients. Out of guideline‐recommended first‐line options, C1‐INH for LTP was reported in 54 patients and lanadelumab in 19 patients.^45^

Conversely, the studies from United States reported established use of first‐line HAE treatment options, with the use of C1‐INH reported in up to 60.7% of patients and the use of lanadelumab (since 2018) reported in up to 53.3% of patients in separate studies.^37^, ^38^, ^39^, ^40^, ^41^, ^42^ Widespread use of C1‐INH was also reported in Canada (93.5% of physicians reported prescribing C1‐INH for LTP),^64^ Germany (ongoing LTP and/or STP with C1‐INH reported in up to 65.0% of patients),^65^ and Puerto Rico (LTP with C1‐INH reported in up to 68.7% patients who were taking prophylaxis).^66^ Despite the adoption of first‐line LTP options, the frequent use of second‐line LTP therapies persisted even in these countries, with 27.2% of patients from the United States reporting the use of androgens for LTP^39^ and 40.0% of patients from Germany reporting the use of androgens for LTP and/or STP.^65^ In a survey of physicians from Canada, 41.9% of physicians reported prescribing androgens and 19.4% reported prescribing antifibrinolytics^64^; in two similar surveys of physicians from the United States, up to 38% of physicians reported currently prescribing androgens and up to 11% of physicians reported currently prescribing antifibrinolytics.^37^, ^38^ These trends are similar to the findings of multinational (Australia, Austria, Canada, France, Germany, Spain, Switzerland, and United Kingdom) study from 2018 which reported that 45.7% of patients overall received LTP with C1‐INH, 34.4% with androgens, and 17.9% with tranexamic acid.^62^

### 2.5 QoL as an increasingly important measure in evaluating treatment outcomes

Complete control of disease – one of the key treatment goals in patients with HAE – can be evaluated by several different measures, including number of attacks, use of on‐demand/rescue medications, number of emergency department visits or hospitalizations, sick leave, and/or activity impairment.^4^, ^7^ Another key goal of HAE treatment is ensuring that patients do not experience limitations caused by HAE symptoms.^4^, ^7^, ^9^ Therefore, monitoring of QoL in patients with HAE has been recommended in the recent clinical guidelines and consensus statements on HAE management.^4^, ^7^, ^9^, ^10^, ^22^, ^27^ In line with these recommendations, recent clinical trials for the HAE‐specific treatments often prospectively evaluate QoL as one of the endpoints.^67^, ^68^, ^69^

QoL can be assessed with patient‐reported outcome (PRO) tools that reflect the benefits valued by the patients.^7^, ^27^, ^70^ PROs can be either generic or disease‐specific; generic PRO tools are less specific and often do not have enough sensitivity for disease‐specific components.^27^ Disease‐specific PROs for angioedema conditions include Angioedema Quality of Life Questionnaire (AE‐QoL), Hereditary Angioedema Quality of Life Questionnaire (HAE‐QoL), Angioedema Activity Score (AAS), and Angioedema Control Test (AECT), with more disease specific tools in development.^27^, ^71^, ^72^, ^73^, ^74^ Furthermore, specific aspects of QoL in HAE can be assessed with non‐disease specific tools, such as Hospital Anxiety and Depression Scale (HADS), 9‐item Treatment Satisfaction Questionnaire for Medication (TSQM‐9), and Work Productivity and Activity Impairment Questionnaire (WPAI).^75^, ^76^, ^77^ However, none of the currently available PROs alone are able to fully assess disease control or patient QoL.^7^ Furthermore, not all of the PROs assessment tools may be available in different languages, thus potentially limiting the comparability of studies done in different countries.^70^, ^78^ Another unmet need is the lack of validated disease‐specific PROs for pediatric patients with HAE (aged <18 years).^7^, ^9^, ^78^

In line with the emphasis that current treatment guidelines and consensus statements have placed on shared decision‐making, QoL assessments should be individualized by including patient input on how disease control and QoL are measured.^4^, ^7^, ^9^, ^27^ The importance of shared decision‐making can be highlighted with a recent survey of patients with HAE and physicians, in which the physicians placed significantly more importance on some of the treatment burdens (eg, HAE medication interfering with daily activities) compared with the patients.^38^

Shared decision‐making is instrumental in developing individualized treatment plans that take account of disease activity as well as the individual patient's life circumstances and preferences.^4^, ^7^, ^9^, ^27^ To support with shared decision‐making in HAE, a 3D (Discover, Discuss, Decide) framework has recently been adapted specifically for use in the HAE setting.^79^ Given the high variability of HAE, such individualized treatment plans are crucial, and the guidelines and consensus statements recommend periodic review of these treatment plans.^4^, ^9^, ^27^ QoL is emphasized as a key factor for consideration in shared decision‐making and individualized treatment plans.^4^, ^9^, ^10^, ^27^

### 2.6 Improvement of QoL in patients with HAE is needed

Overall, the impact of HAE on patient QoL was similar in countries with available data. Impaired QoL in patients with HAE compared with healthy subjects was reported in multiple countries, including the United States,^39^ Canada,^80^, ^81^ China,^53^ and Puerto Rico.^66^ An international study in Australia, Canada, and several European countries also showed decreased QoL in patients with HAE, further reinforcing the consistent effect of HAE on QoL regardless of the country.^62^ Anxiety and depression were reported in the studies from the United States^39^ and Canada^81^ as well as the international study, suggesting a psychological burden for patients with HAE worldwide.^62^ Furthermore, a study in Brazil reported that 79% of investigated patients experienced fear brought by their diagnosis.^82^ Interestingly, there were some country‐specific differences in QoL when only pediatric patients with HAE were considered. Studies in children from Israel and Hungary (QoL reported by child alone or by both child and maternal proxy report using generic Pediatric Quality of Life Inventory [PedsQL] and State and Trait Anxiety Inventory for Children [STAIC] questionnaires) showed impaired QoL and increased anxiety in children with HAE,^83^, ^84^ whereas studies from Sweden (QoL reported by child and/or parent proxy using visual analogue scale [VAS] assessment) and Denmark (QoL reported by child and parent proxy using PedsQL, Children's Dermatology Life Quality Index [CDLQI], VAS assessment, and non‐validated disease specific questionnaire) suggested that QoL in children with HAE was similar to that of healthy children.^60^, ^85^

Another limitation that patients with HAE may experience is a reduction in work productivity. In an international survey, patients reported 25% presenteeism, 24% work productivity loss, and 34% activity impairment.^62^ Similarly, a study from Canada reported 31% absenteeism, 27% work productivity loss, 10% presenteeism, and 21% activity impairment.^81^ In a study from the United States, diminished work productivity was associated with higher numbers of HAE attacks (3.3% work productivity loss in patients who were attack free for 6 months vs. 52.5% in those experiencing ≥13 attacks).^39^ Work productivity impairment, absenteeism, and presenteeism were the highest in attacks that affected the face; activity impairment was highest with abdominal and laryngeal attacks (76.7%).^39^

In addition to interfering with work productivity, disease activity was also reported to affect QoL in patients with HAE. Higher number or higher frequency of HAE attacks were associated with lower QoL in studies conducted in the United States,^39^ Canada,^86^ Hungary,^87^ and Hungary and Israel.^84^ Furthermore, studies from China^53^ and Denmark^85^ showed that QoL was even lower in patients with a recent HAE attack. This is in line with markedly higher burden of HAE during an HAE attack versus attack‐free state as estimated by time trade‐off utility values reported in a study from the United Kingdom.^88^ Therefore, better disease control and corresponding reduction in the number or frequency of HAE attacks may increase QoL, as shown in studies from China^53^ and Germany.^89^ It should, however, be noted that patients with HAE experience QoL impairment between HAE attacks, as well as during an attack: they report ongoing fear of another attack, and disease interference with education and career. Lifestyle modifications to avoid HAE triggers can affect individual patients differently.^27^ Indeed, a study from Hungary reported that some patients with a high number of attacks nevertheless reported only mild QoL impairment; furthermore, the same study also reported that a subgroup of patients with a low number of HAE attacks reported high QoL impairment. These results further highlight the variability of HAE effects on the patients' lives.^87^

As HAE attacks are associated with QoL impairment, a promising avenue to improve QoL in patients with HAE is attack prevention with long‐term prophylaxis. In several clinical trials that investigated prophylactic medications (eg, C1‐INH, lanadelumab) for HAE, prophylaxis was associated with clinically meaningful improvements in QoL.^68^, ^69^, ^90^ Higher QoL in patients who were receiving HAE prophylaxis was also reported in some patient survey‐based studies; for example, in a study from the United States, patients reported improved QoL and mental health as positive effects of their HAE prophylaxis.^41^ In another US study, patients reported higher QoL with any prophylactic treatment versus on‐demand treatment only^43^; and a study from Germany reported significantly better QoL and reduced anxiety and depression in patients who were receiving LTP compared with patients who were receiving on‐demand treatment only.^89^ One of the studies from the United States also reported socioeconomic costs (including lower work productivity, presenteeism, absenteeism, and lower labor market participation) associated with on‐demand treatment only, suggesting that LTP of HAE attacks could be associated with reduction of these costs.^43^ However, some other studies, including another study from the United States,^91^ two studies from Canada,^81^, ^86^ and one study from China^53^ did not find significant improvements in QoL with HAE prophylaxis.

There may be several different reasons explaining why some studies failed to detect QoL improvement with HAE prophylaxis, including features of the studied populations such as actual or perceived burden of HAE treatment, cultural values, severity of disease, as well as the impact on QoL between attacks. Only one study from Canada reported comparison in QoL between patients receiving LTP with different agents (C1‐INH vs. androgens/tranexamic acid/unspecified) as well as those receiving any LTP and those without, and found no differences; however, numerical data for these comparisons were not reported.^86^ In the studies from the United States and Germany that reported higher QoL in patients receiving LTP, 62%–89% of patients who received LTP were using first‐line options (C1‐INH or lanadelumab) for their LTP.^41^, ^43^, ^89^ In contrast, in a study from China that did not detect QoL differences in patients receiving and not receiving HAE prophylaxis, all patients were receiving second‐line option (androgens) for their LTP; other studies reporting no association between LTP and QoL did not report what medications were used for LTP.^53^, ^81^, ^86^, ^91^ As androgen use is often associated with side effects and comorbidities (eg, in a study from Switzerland, 54% of patients who were receiving LTP with danazol and 24% of those who were not reported comorbidities), these could obfuscate the impact of reduced HAE attack rate on QoL and contribute to amplified cost of care.^48^, ^92^

It should be noted that other HAE medications may also be associated with a treatment burden that could be masking the effect of reduced attack number on QoL. In several studies from the United States, patients reported anxiety around taking their HAE medication,^41^ finding needles and/or injections/infusions unpleasant,^38^ and being tired of injections/infusions.^38^, ^41^ However, the majority of patients also reported learning to cope with the difficult aspects of their HAE treatment and being satisfied with their current injectable medications.^40^, ^41^ Additionally, injectable medications for HAE have longer dosing intervals, which have been associated with higher treatment adherence in several different therapeutic areas, presumably due to more patient convenience with less frequent dosing.^93^, ^94^, ^95^

## 3 CONCLUSIONS

Several new treatment options for patients with HAE have entered the market and were included in clinical guidelines in recent years, changing the landscape of HAE treatment and providing patients with more options and empowerment for shared decision‐making. Despite guideline recommendations favoring new HAE‐specific treatments, alternative treatment options such as attenuated androgens and antifibrinolytics continue to be used in daily clinical practice in some geographies, even in countries where the newer treatment options are approved, likely due to relatively easy access and disease control for some patients. This suggests there may be a need for better identification of patients who would benefit the most from the newest treatments. Patient‐reported outcomes and shared decision‐making are emerging as key factors in reducing the burden of HAE and its management. QoL in patients with HAE is important to monitor and depends on many factors, which may include disease activity, disease control, and treatment options used, as well as cultural differences.

## AUTHOR CONTRIBUTIONS

**Anete S. Grumach**: Conceptualization; (Equal), Writing – original draft; (Equal), Writing – review & editing; (Equal). **Noga Gadir**: Conceptualization; (Equal), Writing – original draft; Equal, Writing – review & editing; Equal. **Aharon Kessel**: Conceptualization; (Equal), Writing – original draft; (Equal), Writing – review & editing; (Equal). **Ashley Yegin**: Conceptualization; (Equal). Writing – original draft; (Equal), Writing – review & editing; (Equal). **Inmaculada Martinez‐Saguer**: Conceptualization; (Equal), Writing – original draft; (Equal), Writing – review & editing; (Equal). **Jonathan A. Bernstein**: Conceptualization; (Equal), Writing – original draft; (Equal), Writing – review & editing; (Equal).

## CONFLICT OF INTEREST STATEMENT

ASG has received speaker/consultancy fees from CSL Behring, Multicare, Pharvaris, and Takeda; and a grant of researcher initiative from Takeda.

NG and AY are employees of and hold stock/options in Takeda.

AK has received travel grants from Pharming and Takeda, and honoraria from CSL Behring and Takeda.

IMS has received honoraria, research funding, consultancy fees and travel grants from and/or participated in advisory boards for BioCryst, CSL Behring, Pharming, and Takeda.

JAB has been a clinical investigator for BioCryst, CSL Behring, Ionis, KalVista, Pharming, and Takeda; speaker for BioCryst, CSL Behring, Pharming, and Takeda; consultant for Astria, BioCryst, BioMarin, CSL Behring, Cycle, Ionis, KalVista, ONO, Pharming, Pharvaris, and Takeda; and is an advisory board member of the US Hereditary Angioedema Association.
